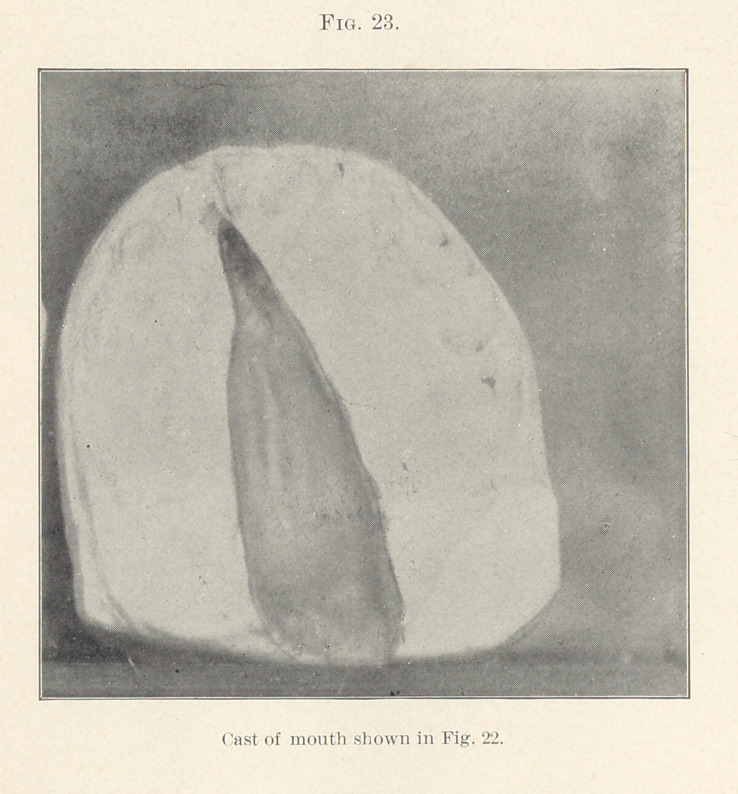# Surgical Correction of Malformation and Speech Defects Due to or Associated with Harelip and Cleft Palate

**Published:** 1902-05

**Authors:** George V. I. Brown

**Affiliations:** Milwaukee, Wis.


### International Dental Journal.

Vol. XXIII. May, 1902. No. 5.

### Original Communications.[1]

### SURGICAL CORRECTION OF MALFORMATION AND SPEECH DEFECTS DUE TO OR ASSOCIATED WITH HARELIP AND CLEFT PALATE.[2]

BY GEORGE V. I. BROWN, A.B., D.D.S., M.D., C.M., MILWAUKEE, WIS.[3]

The purpose of this discussion is to make clear advantageous possibilities in treatment of harelip and cleft palate by a new method which, 1, reduces the width of the fissure and thus renders a subsequent operation for closure by operation upon the soft tissues more certainly successful and more beneficial by preserving the usefulness of the soft parts; 2, by readjustment of the unequally developed bone structures, gives a more perfect contour to the form of the face after operation; 3, makes it possible to operate successfully upon patients almost without regard for age limitations. The objects which make relief of some kind desirable

[1] The editor and publishers are not responsible for the views of authors of papers published in this department, nor for any claim to novelty, or otherwise, that may be made by them. No papers will be received for this department that have appeared in any other journal published in the country.

[2] Read at the fifty-second annual meeting of the American Medical Association, in the Section on Stomatology, and approved for publication by the Executive Committee.

[3] Professor of Oral Surgery, Milwaukee Medical College, and Oral Surgeon, Trinity Hospital.

if not imperative for patients so afflicted, whether the method of procedure be surgical or prosthetic, may be summed up in the two considerations, health and speech, for the purpose of all treatment must be directed towards improvement in one or both of these requisites. Malformations of this nature affect the general health of individuals chiefly in two ways, by malnutrition due to inability of infants to take sufficient nourishment properly, which interferes with normal development sometimes to so great an extent as to place the lives of such children more or less in jeopardy, and nasal catarrh caused by irritating secretions, bacteria, and foreign matter, which gain access to the nasal passages through the opening from the oral cavity or constant exposure of the nasal mucous membrane to external irritants. This diseased condition usually extends to the pharynx and carries in its train many associated disorders that affect the adjacent frontal maxillary and ethmoidal sinuses, involving also nervous and circulatory disorders.

In classifying cleft palate cases, the first division recognized between acquired and congenital cases is important but quite insufficient, and much depends upon a correct and distinct classification of subdivisions under these two heads, which will convey a sufficient understanding not only of the nature of each division, but its relation to the particular kind of treatment required as well, and the special difficulties each presents that must be overcome in effecting a cure. Strangely enough, there seems to be little or nothing especially clear or valuable in this direction to be found in literature upon the subject.

Acquired cases have one of two etiological factors,—disease or accident. When the tissues of either soft or hard palate are destroyed by pathological condition, naturally the advisability or inadvisability of an operation would be determined by the nature of the cause. For instance, in syphilitic cases operation would usually be contraindicated, because of the tendency to still further loss instead of restoration of tissue. If the opening be confined to the hard palate, as frequently occurs from necrosis, such an opening is much more easily and better covered by a nicely fitted denture, but if the tissue of the soft palate has become involved, speech is impaired to such a degree that more or less risk is warranted in the hope of restoring the ability to speak distinctly. Classification of congenital cases presents difficulties which can best be understood by considering them with regard to the nature of the deformity, since there are such notable differences between typical forms of congenital fissures of the palate and lip, each with its own characteristic difficulties to be overcome in treatment, and again, in order that the surgeon may intelligently undertake their correction, it is necessary that another distinction be made with regard for the vital question of age in relation to operative procedures. Under 1, we distinguish clefts in the soft palate with hard palate normal; 2, a continuous separation through both hard and soft palates; 3, double cleft, which may bifurcate from a single one at the intermaxillary bone, or may make two continuous fissures through hard and soft palates. Age requires recognition by the following divisions representing distinct operative differences: 1, infants for whom immediate operation may become necessary on account of inability to obtain proper nourishment; 2, eight to ten months old; 3, after the deciduous teeth have erupted but before habits of speech are fully established; 4, older children and adults after the eruption of permanent teeth.

### HIGH MORTALITY IN INFANCY.

The danger and high rate of mortality recorded after infants have been operated upon are too well understood among surgeons to warrant an extended discussion, for whether the death-rate be fifty per cent., as given by Ehrman, or something less, as stated by Schied, Wolff, Fowler, and other writers, it nevertheless goes without saying that if good results can be obtained by operation performed when the child has gathered power of resistance, the method which can accomplish this result must be the better method. So long as it was claimed that, in order to have perfect union of the parts and anything like a perfect development of speech afterwards, it was absolutely essential that the operation be performed in early infancy, such operations might then be excusable, but while it must be admitted that infants take chloroform with comparatively little danger, and do not suffer shock through fear of danger the nature of which they are unable to comprehend, it is only too true that they bear the loss of blood badly. In such cases, with the mouth full of wires and sutures, the inducement to take nourishment through the mouth is not very great. In fact, rectal feeding must almost invariably be resorted to because the digestive tract suffers more or less disturbance by bacteria, from the wound surfaces and in various ways. If nourishment per rectum can be continued and vitality of the child sustained until the stomach and intestinal tract are sufficiently restored to perform their normal functions, success might reasonably be expected, but too often irritation of the rectum interferes before the activity of the digestive organs is restored; then and frequently does the prognosis become exceedingly grave.

### CHILDREN AGED SEVEN TO EIGHT MONTHS.

When the child has passed through the first seven or eight months of infancy and is upon a fairly secure vital basis, the treatment preparatory to a future operation for harelip can best be given by the method shown in Fig. 1, and the reasons why such preparatory treatment is necessary by Figs. 1, 2, 3, 4, and 5, which portray the unequal development that is invariably found in these cases. If no such preparatory treatment be given, there will be a less perfectly shaped mouth, because of the extreme tension due to an effort to make the tissue bridge a wide space, and flatness of the mouth, because of the fact that there is no bone structure behind it, also a flatness of the ala of the nose upon the affected side, with deflection of the nasal cartilage to the opposite side, giving a markedly irregular appearance to nose, lip, and face. This is due to the fact, very clearly noticeable in the pictures referred to, that arrest of development of the jaw upon one side or the other causes a decidedly uneven appearance to the face. Since on one side the bones of the palate protrude, while upon the others there is a corresponding recession. To overcome this a heavy wire suture is passed from the buccal surface upon one side directly through both portions of the jaw to the buccal surface of the other, drawn closely and fastened at each side with little silver plates to prevent it pulling through. (See Fig. 1.) The direction of this wire is governed in such a way that when it is slightly twisted in the centre of the mouth from day to day the tension will bring forward one side and draw back the other. This force, while gently and regularly exerted, is sufficient to alter the form of the bones in any direction desired, at the same time it brings the two sides of the cleft nearer together and reduces the difficulty of future operation upon the palate quite materially. When proper form of adjustment has been secured in this manner, operation for harelip can be performed very easily and perfectly, because the space to be covered has been reduced to the minimum. The nose and bones of the face are thus straightened, and in all respects the result is more perfect than could otherwise have been accomplished. This is shown in Figs. 4, 5, and 6, which are pictures of the same baby and cast of his mouth before operation. The wires can then be removed, because the tension of the lip muscles will continue to exert a force, which will prevent widening of the cleft in the palate that would otherwise take place and continue to narrow it. Thus the operation of uranorrhaphy and staphylorrhaphy become much simplified and can be performed at any period that may seem most favorable to the circumstances and condition of the patient. Figs. 7, 8, and 9 portray in some degree the appearance of a child for whom the unopposed muscles had pulled the detached intermaxillary bone until it stood out in such a way as to appear to be a continuation of the nose, from the end of which two teeth were being erupted. In this case a little more than an inch of the projecting bone was resected, the borders of the jawbone on each side freshened, the intermaxillary forced back to its natural position and wired to the lateral portion of the jaws; operation for double harelip was then performed, with the result shown in Fig. 10. By removing a section from the middle of the bone instead of cutting off at the end to restore the defective form of face and lip, the erupting deciduous teeth, together with the germs of permanent ones, were preserved, and we know, as the child becomes older, development of these teeth will give to face and jaws an almost perfect contour instead of the deformed appearance which would otherwise have resulted.

### CHILDREN WITH DECIDUOUS TEETH.

The care of cases of the third class is illustrated by Fig. 11. In these the deciduous teeth form attachments, to which are cemented metal bands that hold the appliance, which consists of a nut and bar with a thread cut upon it, so that all the different parts are brought into place by turning the nut slightly several times a day, such a pressure being brought to bear that the parts upon each side are drawn towards each other. In children of this age the bones yield readily, and in a few days, without pain or serious inconvenience to the child, the two sides of the cleft can be approximated so closely that when a bur in a surgical engine is passed along between the two borders it will cut off the soft tissue and also freshen the borders of the bone. The parts can then be screwed tightly together and the hard palate given opportunity to unite without fear of sloughing, with the result that a complete bony union is secured, which in most cases can be depended upon to include all that portion of the cleft from the first deciduous molars forward. The benefit of this is apparent because of more complete circulation, which renders the later operation to be performed for closure of the posterior portion of the cleft and the soft palate much less likely to be unsuccessful than it would be under other circumstances. Besides this, the space is so much narrower than would otherwise be the case, that the operation is in all respects so simple as to make a good result comparatively certain. Again, with the bones united, we know that after the permanent teeth are erupted an orthodontia appliance which will exert pressure in exactly the reverse direction from the one used in reducing the cleft will widen the arch again to normal and even perfect form. Fig. 12 shows No. 11 after operation. Fig. 13 shows the case of a little boy who had been operated upon five or six different times unsuccessfully by other methods, but whose palate fissure was easily reduced by this method in a very short time.

### ADULTS AND CHILDREN WITH PERMANENT TEETH.

The treatment of cases of the fourth class—shown in Figs. 14, 15, 16, and 17, all of the same case—is in all respects practically the same, except that the appliance needs to be made of strong material, because permanent teeth form points of attachment and older jaws give more resistance. When it is desired to hasten the contracting process, the patient is anæsthetized and the surgical engine bur passed along the posterior portion of the buccal side of the jaw just under the teeth and the external plate of the jawbone cut through, this being the point at which the greatest resistance is offered, and when both sides have been weakened in this manner pressure is made by the use of suitably adjusted forceps, which bring the two sides as nearly together as possible without complete fracture. The appliance and screw hold the parts in place, and continuous turning of the nut quickly brings the parts in close approximation from the bicuspids forward, altering the occlusion of the jaws so that upper molars, instead of occluding with buccal cusps outside the buccal cusps of inferior molars, will meet upon the inside. The width of a molar tooth can be taken from the width of the space between the bones at the fissure without interfering with the proper performance of the function of mastication, and all who are familiar by practical experience with cases of this character will readily understand what a considerable difference the width of a molar tooth, less space to cover, must be in relation to successful results, so far as securing a covering is concerned, and also for having less tension of soft palate tissue in the effort of speech. The principles of this method have long been practised by orthopædic surgeons in the treatment of malformation of other bones and by dentists in correction of irregularities of the dental arch. What is claimed for the method outlined is that for the first time systematic application of all these principles has been combined with methods of surgical procedure which make it possible to operate with comparatively complete success upon all cases without regard to age or the nature of the cleft, provided there be no serious physical bar to operation. If, then, this can be done at practically any age, it becomes important for us to consider all these matters in relation to results from the stand-point of acquiring perfect speech. It naturally follows that, having thus supplied a covering for the roof of the mouth of living, healthful tissue, and the same for the soft palate, a marked improvement in speech would be expected, yet this is not so, and the question as to "why it is not so" embodies in itself many considerations. While flexibility of the velum after operation and absence therefrom of scars with cicatricial tissue to stiffen and to interfere with the muscular movements are desirable, even indispensable for its perfect vocal assistance, the real reason why the speech of adults improves so little is the same as that which causes an American to find it difficult to speak French, German, or other foreign language, and when he does so to think the sounds he makes the same as those made by natives in pronouncing the same words, for he is conscious of no distinguishing difference, whereas differences do exist in a marked degree and are quite noticeable to other people. There are several elements in the explanation of this fact that are worthy of consideration and lead us directly to a study of the mechanism of speech. To get perfect speech as a result of palate operation we must, 1, supply tissue, which will serve to prevent the nasal sound by shutting off the nasal passages at the proper time; 2, the ear must become so trained as to distinguish readily incorrect sounds in the pronunciation of words; 3, the brain-centres which receive the impressions that excite sound vibrations must carry to the motor centres messages that will set the right muscles in action in the right way. Invariably with older patients is it found that efforts to speak in spite of cleft-palate deformity have caused greater development of muscles, the application of which are usually a hinderance instead of a benefit.

### SPEECH-CENTRES.

The so-called area of speech, as commonly conceded, involves something more than a distinctly outlined portion of the brain-structure, but in a general way it may be understood that in and about the Sylvian fissure, the third frontal convolution, the Rolandic area, and that particular portion known as Broca's convolution, the various psychomotor centres necessary to vocalization are located, and a consideration of the association of these parts to each other will convince the careful reasoner that in the correction of these oral deformities there are many difficulties to be considered besides merely the task of securing an adjustable veil which will serve to close or open certain air-passages during the act of speech as in the normal individual. A study of the brain-structure and of the principles upon which modern brain surgery is based, proved over and over again through experiments upon lower animals and by clinical experience in the study of aphasia and other affections of speech due to diseased conditions, makes clear the fact that the perfect formation of words and their construction into intelligent sentences requires the co-operation of many distinctly different nerve-centres.

The first cry of the child is merely a sound caused by reflex action of the muscles without any guiding influence exerted by the faculty of reason. This is followed by the first efforts of sound-making to represent intelligent words as objects begin to be recognized, and gradually this is continued and extended until expression of ideas in speech has become possible. It will be readily understood that, if these efforts have been upon normal or correct lines, the muscular activity necessary to sound-producing must have been guided by the proper nerve-centres, which will have caused an increase in the brain development of those centres, and the messages sent from the motor tract to the muscles which are concerned in the utterance of words will be in all respects correct; therefore, the habit of proper speech will have become an established fact; but, on the other hand, if a deformity has existed from birth by reason of which the normal use of certain muscles will have been greatly restricted and the use of certain other muscles not commonly used in the process of word enunciation, it will have received more stimulation than would have been the case had there been a perfectly formed mouth and throat; the result must invariably be an increased development of the nerve-centres which are injurious to speech, with a faulty development of those that are necessary to perfect speech. This would be termed a habit, but the word habit conveys too restricted an idea of the condition. For example, when the eye through its retinal image registers upon the brain-structures the particular nerve stimulations which in time shall become associated with the name of an object, its form record is established by what may be known as the visual memory centres. In the same manner the sound of the name of the subject through vibrations of the auditory nerves and organs of hearing becomes fixed in the auditory memory centres. Precisely also are the somæsthetic areas affected by the tactile sense, and memory of the sense of touch, as well as taste, smell, or other stimulus that may have been excited by or associated with any particular object, and when the sensorium takes consciousness of this object, the name of which has become known to it, there is required the co-ordination between these different memory centres in order that the proper messages may be sent to the motor centres through which certain muscles may be set in motion in the proper manner to produce the sound which may be clearly recognized as the spoken name of the object.

It is known that in speech the muscles of the chest which are responsible for expiration, the muscles that raise and lower the larynx, those that tighten the vocal cords and tip the hyoid bone, as well as resounding properties due to the nearness of the spinal column, and the co-operation of the forces that are applied in raising and lowering the soft palate, the adjustment of the tongue, proper action of the muscles of the cheeks and lips are all necessary for utterance of even a single word. If, therefore, during the life of the individual, through faulty operation or adverse action of these agencies, wrong messages have been constantly sent to any portion of the brain concerned in making a certain sound, and if the auditory memory centres have registered by the constant hearing imperfect sounds for specific words, which will accordingly have caused the development of brain-structure that is all active against correct speech, and if there be an insufficient development of those centres which are needed for perfect speech, how great becomes the difficulty of giving a speech power to individuals in the face of all these acquired disadvantages!

It is too narrow a view of his own special portion of the sound-making apparatus that has so limited the oral surgeon in his consideration of this subject in the treatment of cleft palate. There has been laid down the absolute necessity of having a soft palate which will be capable of entirely excluding the passage of the air to the posterior nares, thus to overcome nasal tones, but a well-known writer has recently stated that this is a false idea, since there are many times in speaking and singing, during which the passage of the air through the nares is not excluded, and yet no nasal tone is noticeable. As a matter of fact, both the quality and tone are largely decided before the sound wave has reached the soft palate.

Practical examples of the truth of these theoretical principles are shown by the accompanying illustrations. Figs. 18, 19, 20, and 21 show photographs and casts of the mouth of a patient before and after operation. The cleft was confined to the soft palate alone.

Figs. 22 and 23 show a very large cleft extending through both hard and soft palates. Both these patients are girls about the same age, yet the speech of No. 22, with a much greater deformity, was somewhat clearer than No. 18 before operation, with a smaller opening in the palate. This is particularly interesting because an unusual development of the muscles that raise the tongue and constrictors of the larynx had enabled No. 22 to force the back of the tongue up into the cleft and narrow the pharyngeal opening, so as to overcome, in a measure at least, the deficiency, but the unusual muscular action will undoubtedly militate against success after operation when the case is completed. No. 18, having been given almost a perfect velum (see Figs. 19 and 21), one quite flexible and free from the thickening of scar tissue, while much improved in ordinary conversation, retains a good deal of the characteristic disagreeable vocal sounds, and when excited becomes almost unintelligible, yet when reciting a little piece that I have recently taught her to say properly, the involuntary office of wrong auditory and other memories being by effort excluded, she has nearly perfect vocalization, marred only by a slight nasal tone not more than many persons with normal palates acquire through

Fig. 17.

Cast of mouth of same case as Figs. 14, 15, and 16, after operation.

Fig. 18.

Girl sixteen years old: cleft soft palate.

Fig. 19.

Same case as Fig. 18, after operation.

Fig. 20.

Cast of mouth of Fig. 18, before operation.

Fig. 21.

Cast of mouth of same case as Figs. 18, 19, and 20, after operation.

Fig. 22.

Girl sixteen years old : cleft through both hard and soft palates.

Fig. 23.

Cast of mouth shown in Fig. 22.

catarrh or habit, and thus we have clearly established the fact that only a few weeks after operation it is possible with a little care to get nearly perfect speech. A summary of conclusions from the foregoing would be as follows:

1. The risk of operation in early infancy is unnecessary except where vitality of the child is threatened by malformation.

2. The most favorable time for operation is after the deciduous teeth have been erupted, but before the habit of speech has been acquired.

3. Difficulty of acquiring correct methods of pronouncing words after operation in adult cases can only be overcome by careful mental training.

4. There can be no cases which cannot be improved by treatment and operation, both with regard to health and speech, no matter what the age may be, providing the co-operation and assistance of the patient may be assured.

## Figures and Tables

**Fig. 1. f1:**
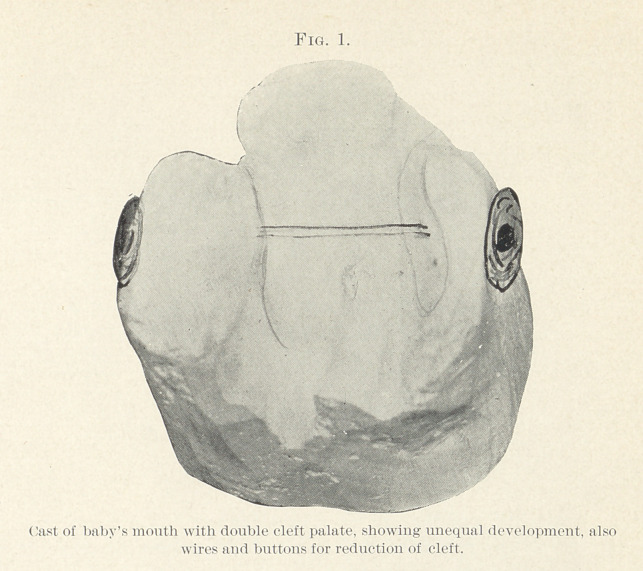


**Fig. 2. f2:**
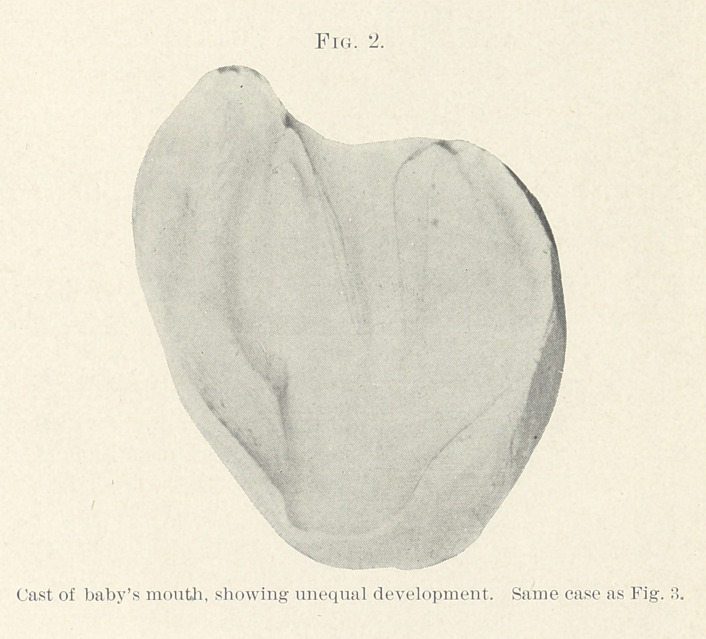


**Fig. 3. f3:**
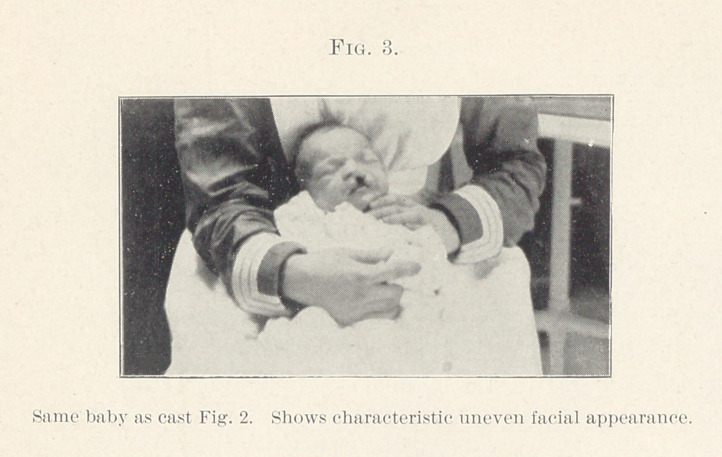


**Fig. 4. f4:**
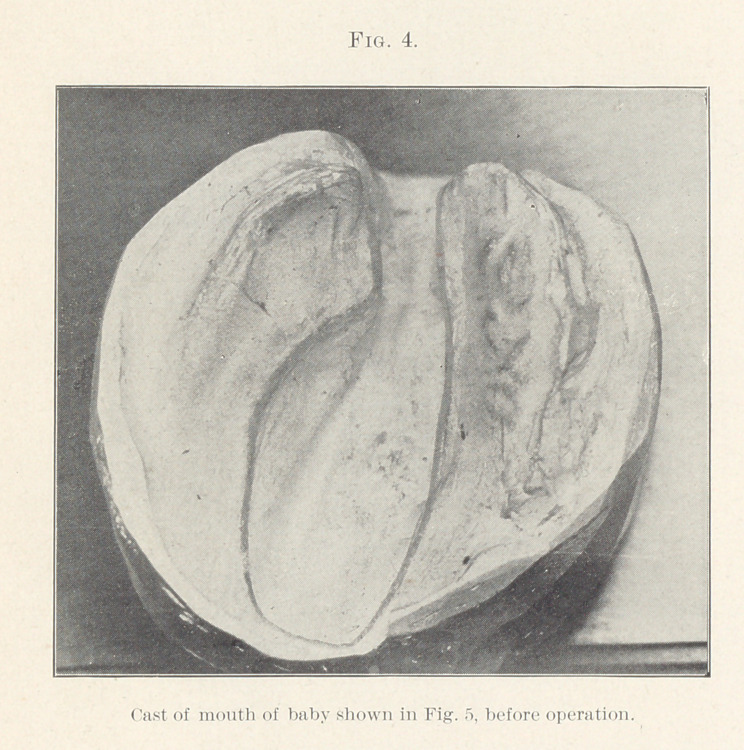


**Fig. 5. f5:**
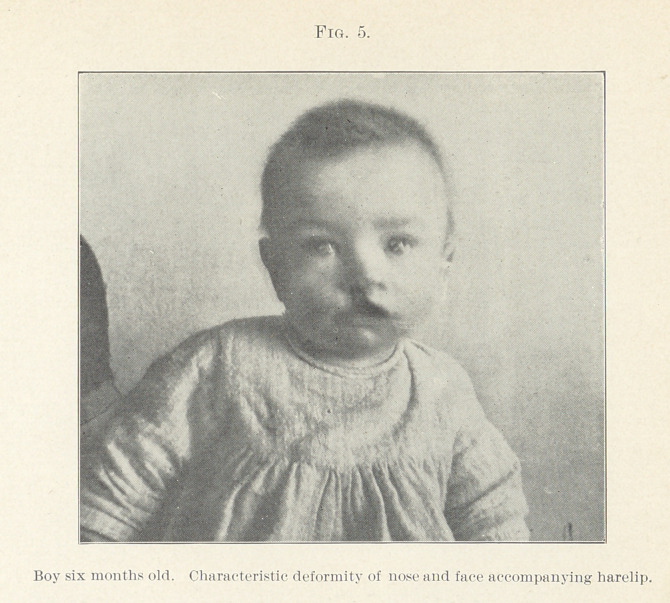


**Fig. 6. f6:**
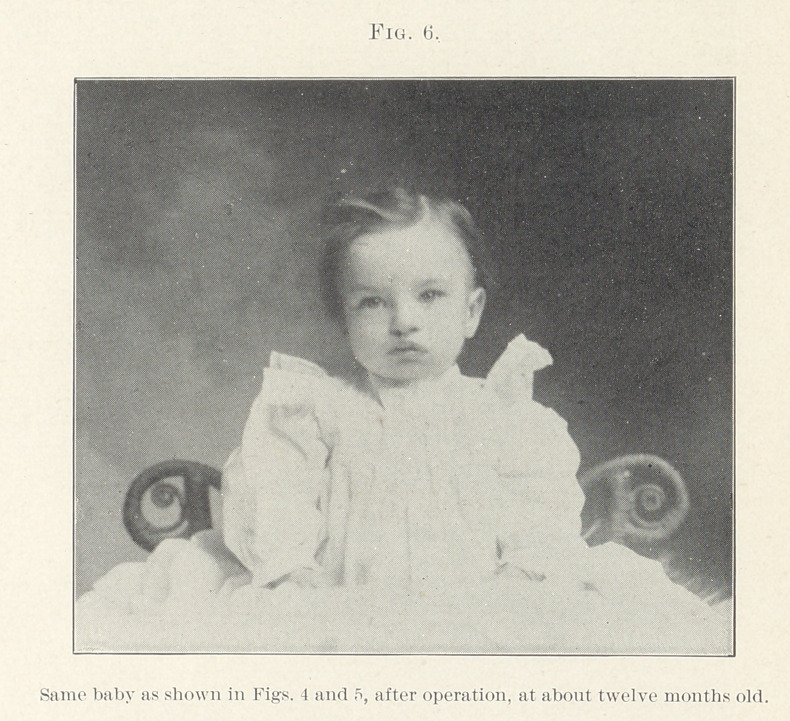


**Fig. 7. f7:**
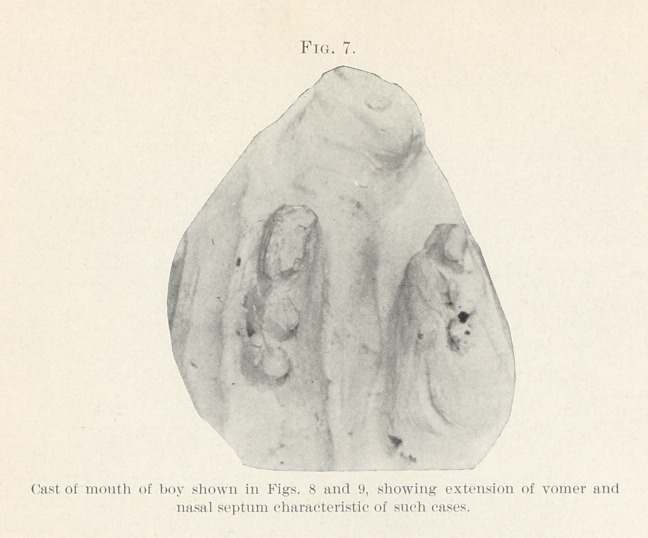


**Fig. 8. f8:**
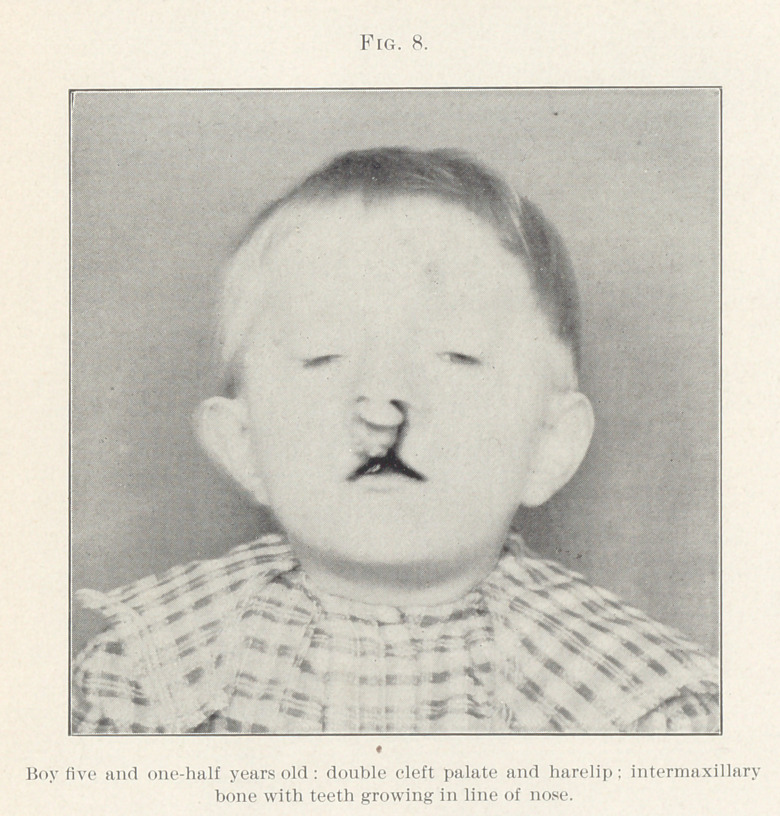


**Fig. 9. f9:**
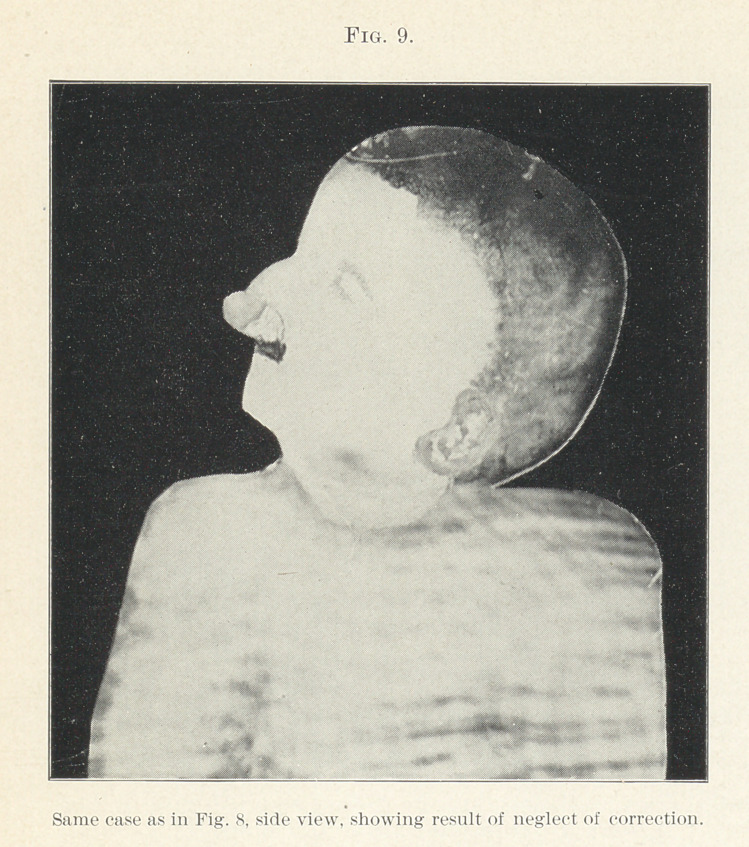


**Fig. 10. f10:**
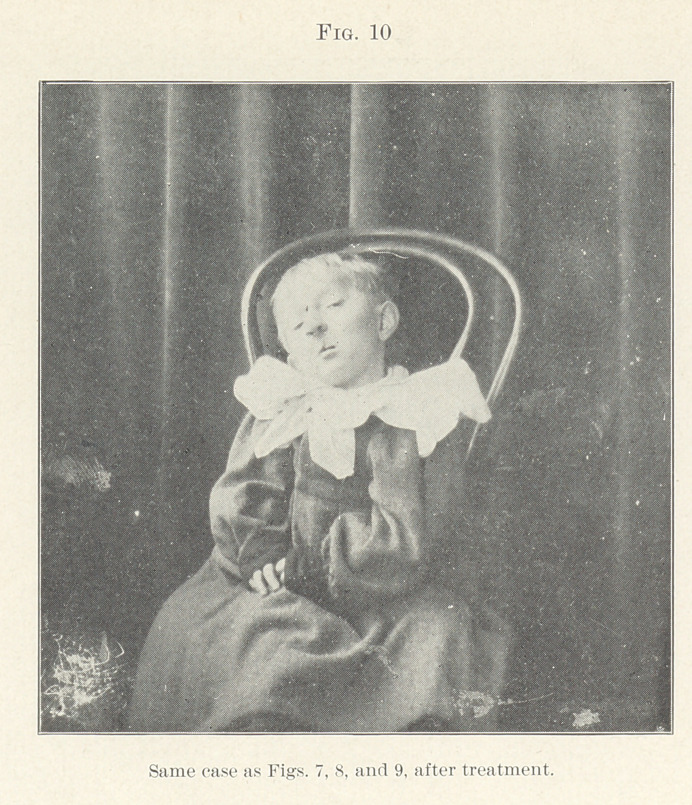


**Fig. 11. f11:**
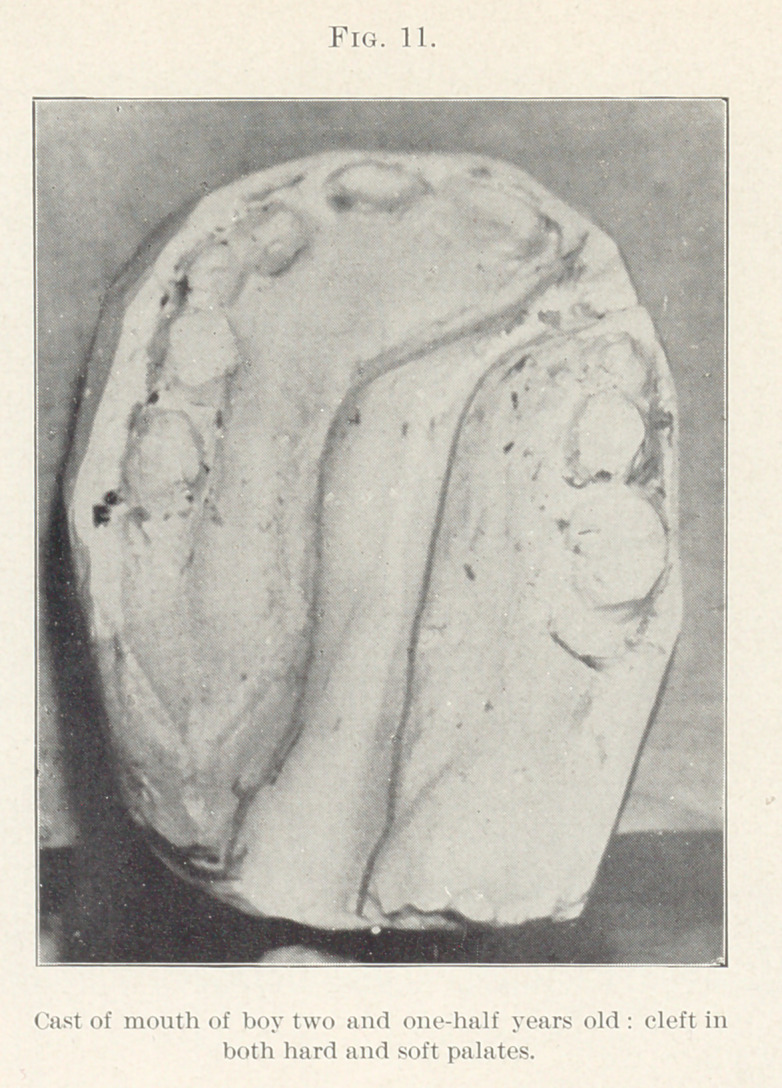


**Fig. 12. f12:**
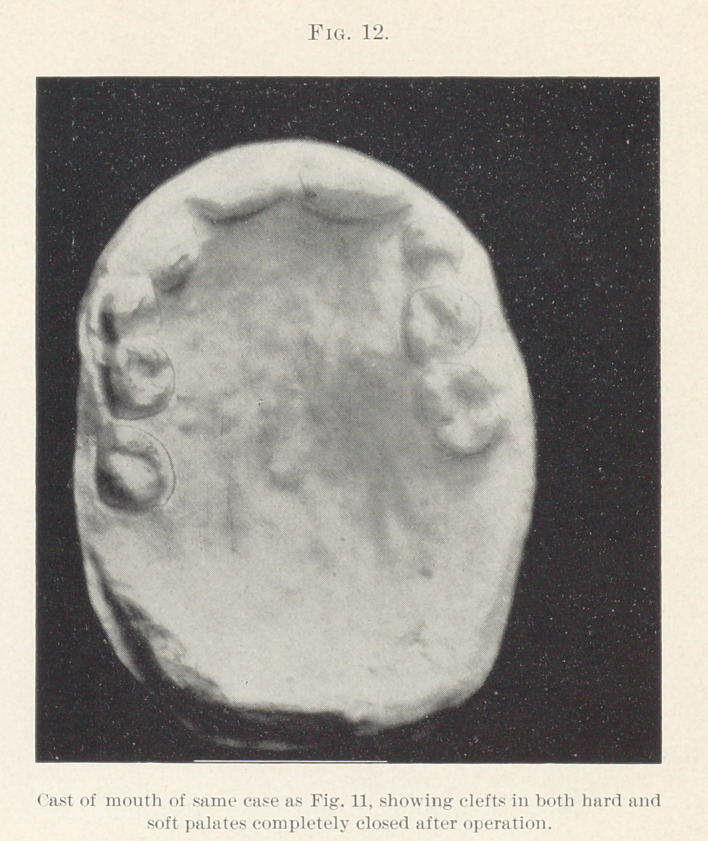


**Fig. 13. f13:**
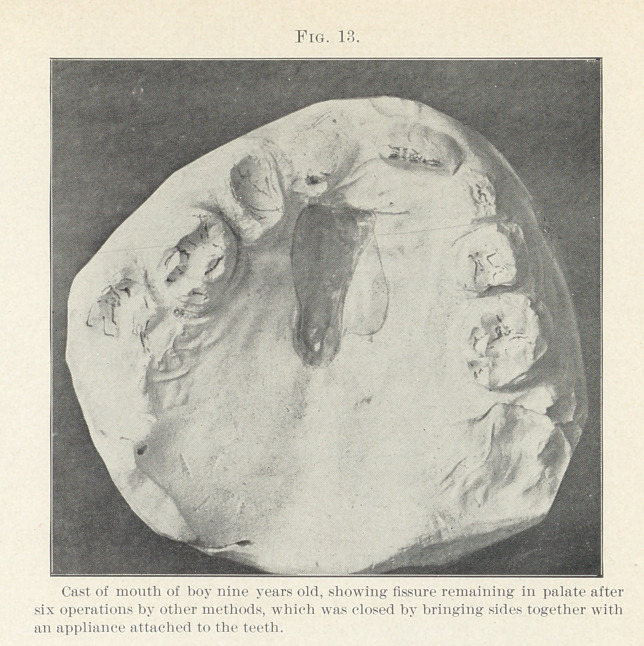


**Fig. 14. f14:**
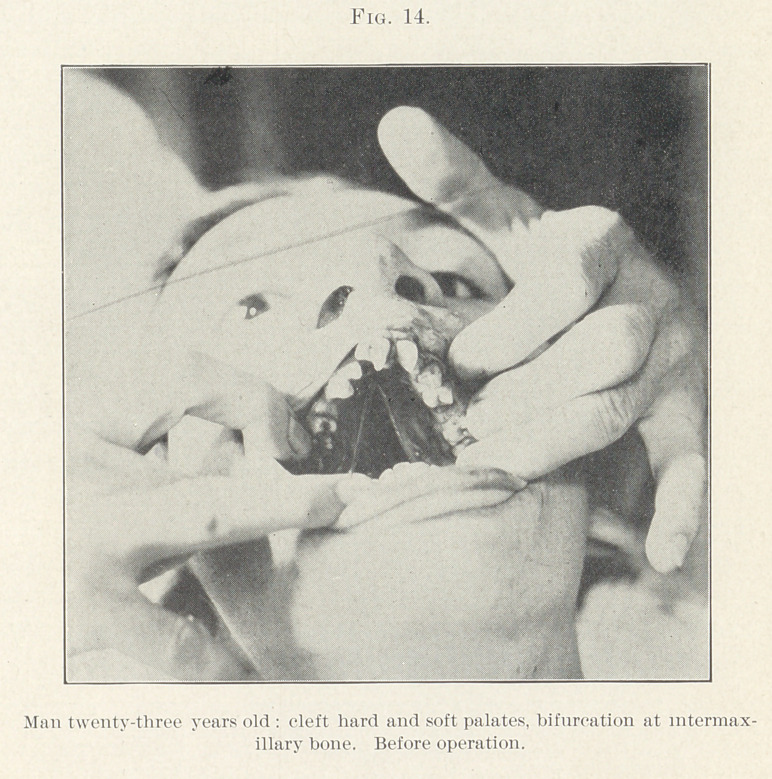


**Fig. 15. f15:**
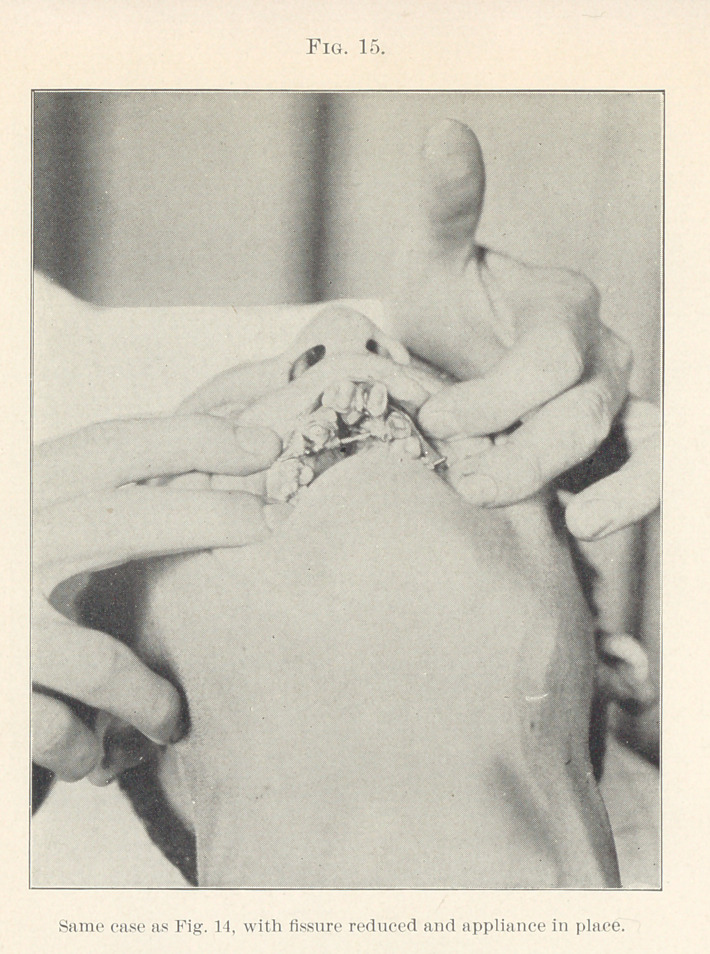


**Fig. 16. f16:**
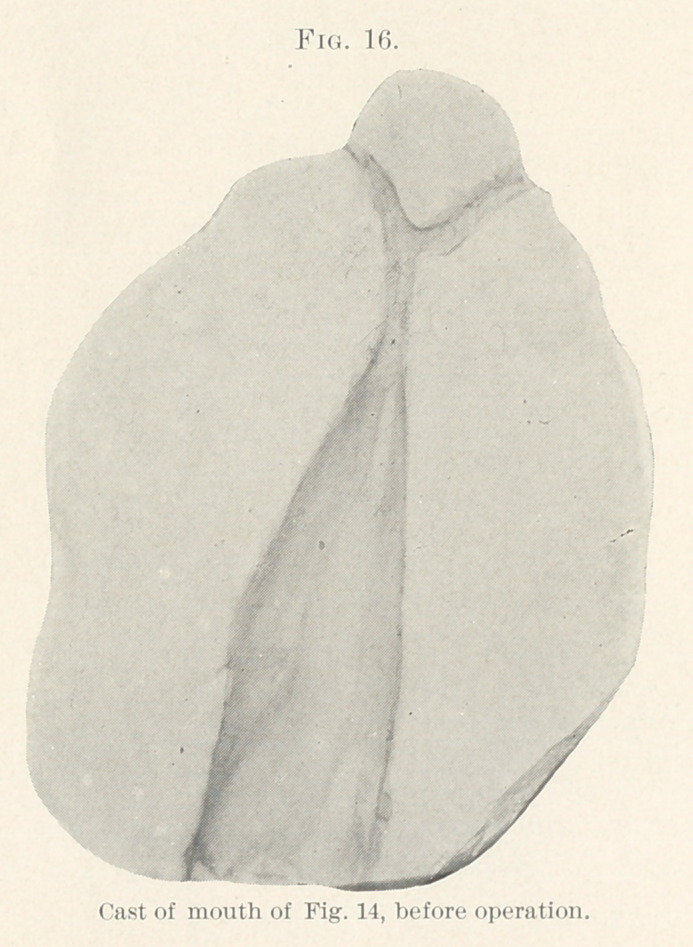


**Fig. 17. f17:**
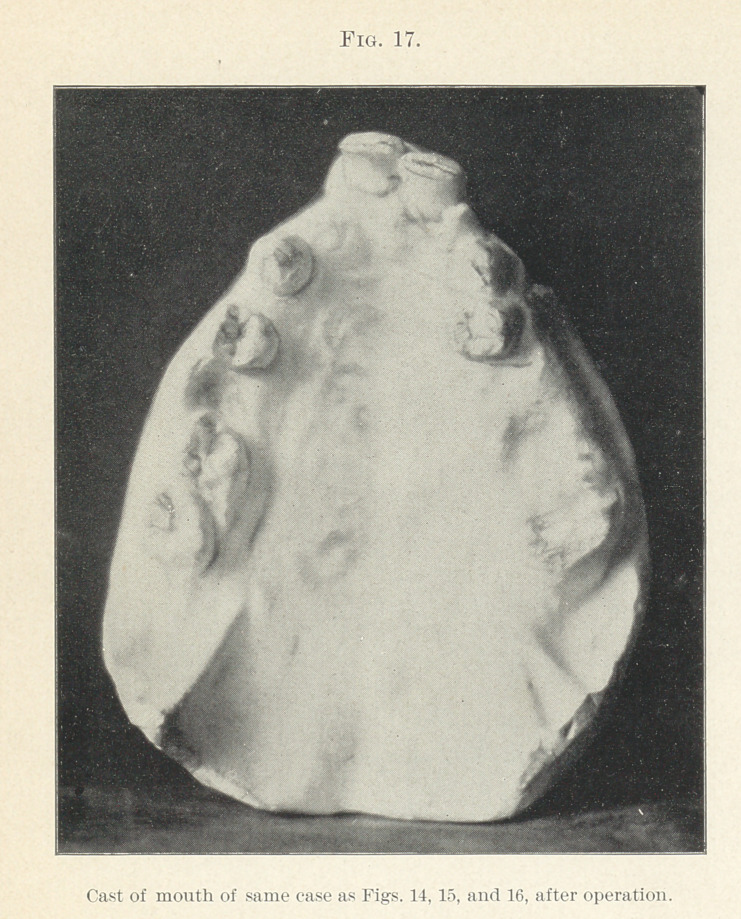


**Fig. 18. f18:**
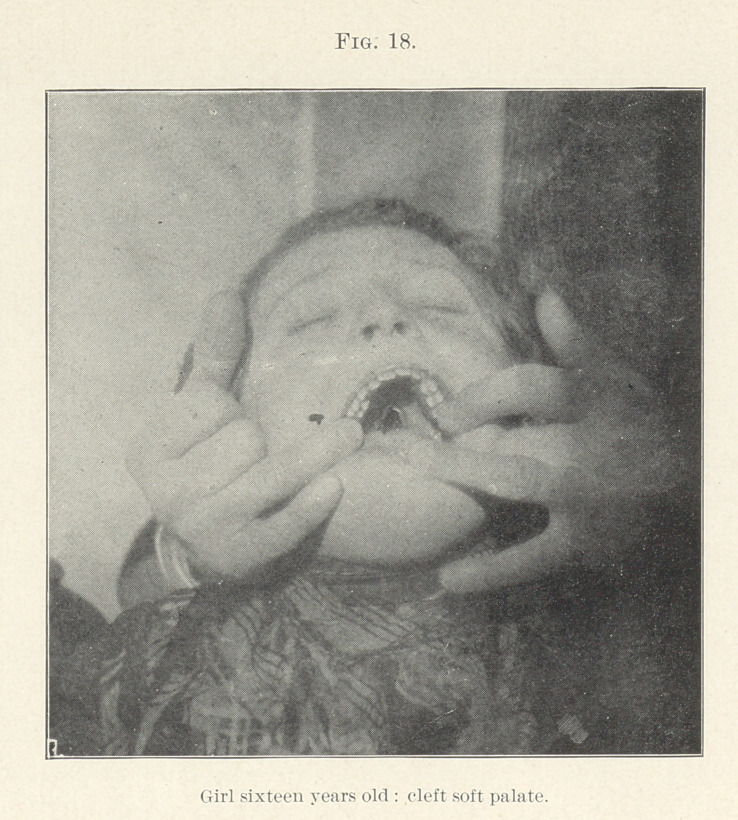


**Fig. 19. f19:**
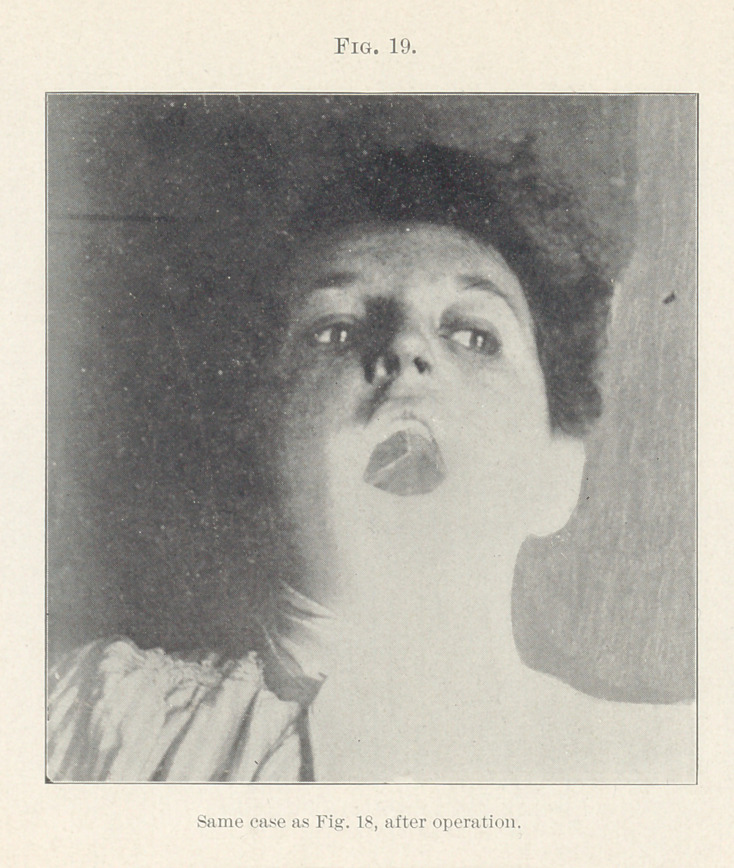


**Fig. 20. f20:**
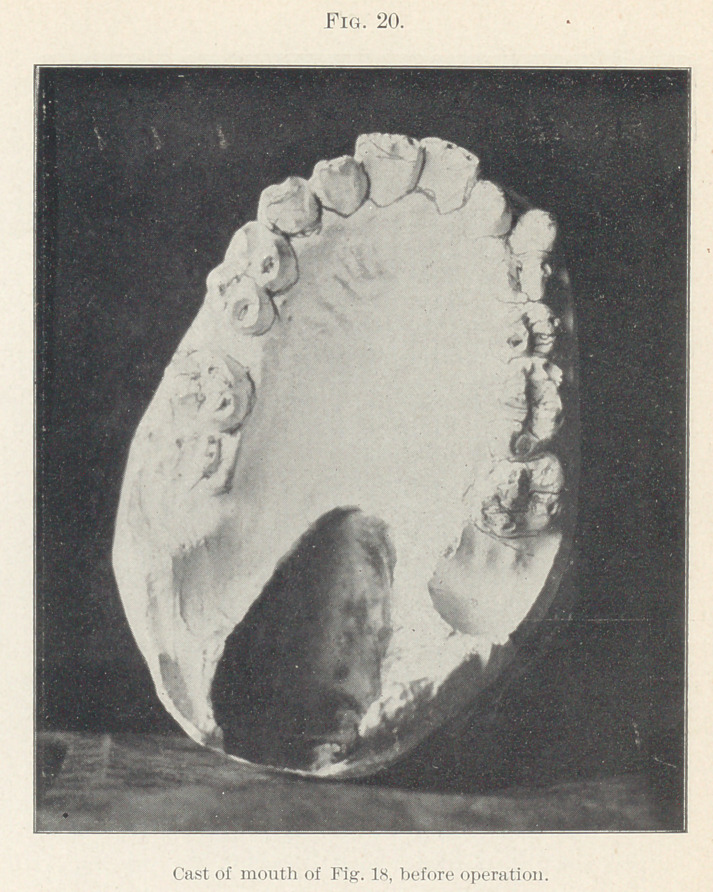


**Fig. 21. f21:**
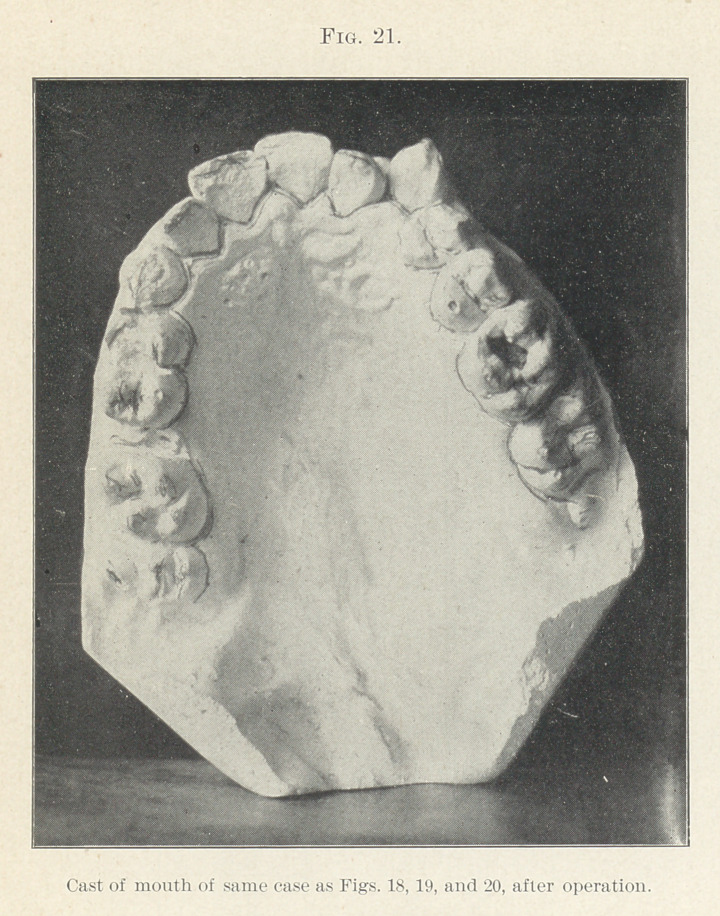


**Fig. 22. f22:**
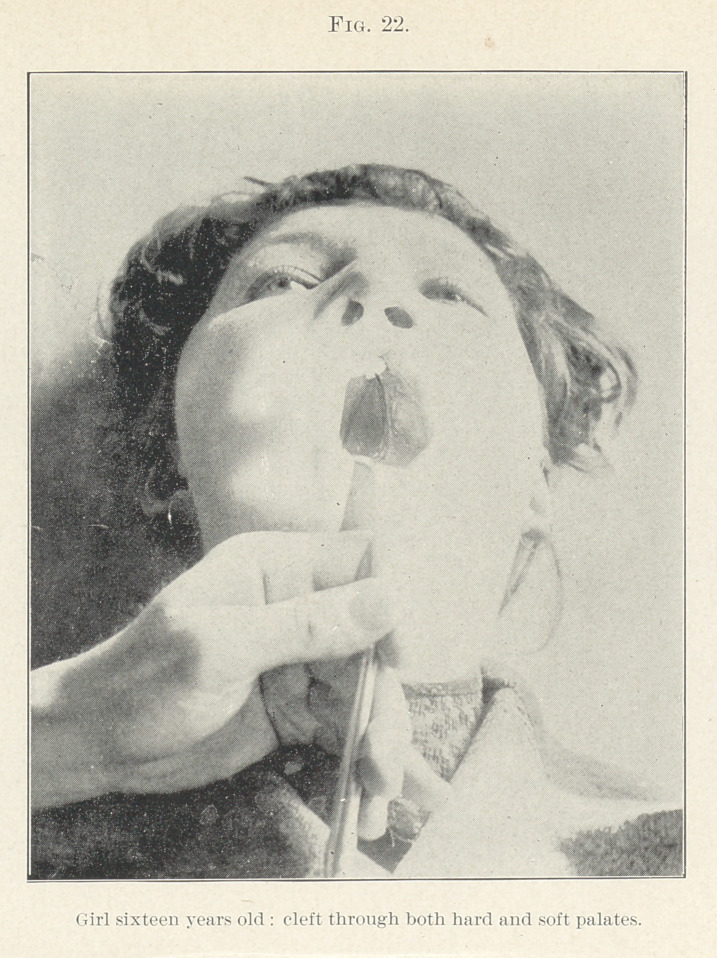


**Fig. 23. f23:**